# Synergistic Toxicity of Plant Essential Oils Combined with Pyrethroid Insecticides against Blow Flies and the House Fly

**DOI:** 10.3390/insects10060178

**Published:** 2019-06-21

**Authors:** Suttida Suwannayod, Kabkaew L. Sukontason, Benjawan Pitasawat, Anuluck Junkum, Kwankamol Limsopatham, Malcolm K. Jones, Pradya Somboon, Ratana Leksomboon, Theeraphap Chareonviriyaphap, Apiwat Tawatsin, Usavadee Thavara, Kom Sukontason

**Affiliations:** 1 Department of Parasitology, Faculty of Medicine, Chiang Mai University, Chiang Mai 50200, Thailand; suttida294@gmail.com (S.S.); kabkaew.s@cmu.ac.th (K.L.S.); benjawan.p@cmu.ac.th (B.P.); anuluck.j@cmu.ac.th (A.J.); kwankamol.l@cmu.ac.th (K.L.); pradya.somboon@cmu.ac.th (P.S.); 2 Graduate School, Chiang Mai University, Chiang Mai 50200, Thailand; 3 School of Veterinary Sciences, The University of Queensland, Gatton 4343, Australia; m.jones@uq.edu.au; 4 College of Medicine and Public Health, Ubon Ratchathani University, Ubon Ratchathani 34190, Thailand; ratana_tlek@yahoo.com; 5 Department of Entomology, Faculty of Agriculture, Kasetsart University, Bangkok 10900, Thailand; faasthc@ku.ac.th; 6 Department of Medical Sciences, Ministry of Public Health, Nonthaburi 11000, Thailand; apiwat@health.moph.go.th (A.T.); usavadee99@gmail.com (U.T.)

**Keywords:** plant, essential oil, pyrethroid, synergism, fly control

## Abstract

Blow flies (Diptera: Calliphoridae) and the house fly (Diptera: Muscidae) are filth flies of medical importance, and control of their population is needed. As insecticide applications have resulted in fly resistance, and the exploration of plant essential oils (EOs) has increased against filth flies, this study assessed the combination of EOs with pyrethoids to enhance toxic efficacy. The EOs of five effective plants were screened initially against the house fly (*Musca domestica* L.). Their chemical constituent was performed using gas chromatography-mass spectrometry (GC-MS) analysis. The main components of *Boesenbergia rotunda* (Zingiberaceae) rhizome, *Curcuma longa* (Zingiberaceae) rhizome, *Citrus hystrix* (Rutaceae) fruit peel, *Ocimum gratissimum* (Lamiaceae) seed, and *Zanthoxylum limonella* (Rutaceae) fruit were δ-3-caren (35.25%), β-turmerone (51.68%), β-pinene (26.56%), p-cumic aldehyde (58.21%), and dipentene (60.22%), respectively. The screening test revealed that the three most effective plant EOs were from *B. rotunda*, *C. longa* and *O. gratissimum*, which were selected for the combination with two pyrethroid insecticides (permethrin and deltamethrin), in order to enhance their synergistic efficacy against the blow flies, *Chrysomya* *megacephala* Fabricius, *Chrysomya* *rufifacies* Macquart, and *Lucilia cuprina* Wiedemann, and the house fly. Synergistic action was presented in almost all of the flies tested with permenthrin/deltamethrin/EOs mixtures. It was interesting that the combination of deltamethrin with three EOs showed a synergistic effect on all of the tested flies. However, an antagonistic effect was observed in *C. megacephala* and *M. domestica* treated with permethrin-*B. rotunda* and *C. megacephala* treated with permethrin-*O. gratissimum*. The LD_50_ of insecticides decreased when combined with plant EOs. This alternative strategy will be helpful in developing a formula for effective fly control management.

## 1. Introduction

The blow flies, *Chrysomya megacephala*, *Chrysomya rufifacies* and *Lucilia cuprina*, and house fly, *M. domestica,* are recognized as medically important pests worldwide, including Thailand. A systematic survey using a semi-automatic trap throughout one year in Chiang Mai province, Northern Thailand, indicated that these four species accounted for 96.3% of total flies captured in variable areas. A high number of these species was captured throughout the year, with a peak population in the summer [1]. Adults breed and feed on decaying matter, human waste, and food, thereby transmitting infectious pathogens (e.g., viruses, bacteria, protozoa, helminth eggs) from filth substrates to human food that can cause illness and/or diseases [2,3,4,5]. The nuisance capacity of adult flies to humans and agronomic livestock is included. Not only the adults produce effects, but also the larvae, particularly *L. cuprina*, which cause myiasis in agronomic livestock, leading to economic losses [6].

Due to the negative impact produced by *C. megacephala*, *C, rufifacies, L. cuprina* and *M. domestica*, population control is needed, in order to reduce the risk of contamination, and minimize their nuisance capacity. The approach to fly control involves chemical treatments through the use of synthetic insecticides. In Northern Thailand, long-term usage of insecticides has resulted in susceptible strains of these species developing increased resistance, as detected with permethrin and deltamethrin [7,8].

Increasing challenges in using biopesticides for fly population management include exploring plant essential oils (EOs) for reducing the use of chemical insecticides. It is interesting that the volatile substance of EOs is rich with monoterpenes that defend plants from herbivores and pathogens by blocking predation, deterring oviposition, inhibiting growth, and repelling and mimicking juvenile hormones [9,10]. Furthermore, plant extracts are eco-friendly, biodegradable, pest-specific, and safe for mammals [11,12]. Previous studies revealed that a range of plant EOs, including *Eucalyptus globulus* [13], *Mentha piperita*, *Zingiber officinalis*, *Emblica officinalis*, *Cinnamomum verum* [14], *Cymbopogon citratus*, *M. piperita,* and *Lavandula angustifolia* [15] were toxic to *M. domestica*. Likewise, larvicidal and adulticidal toxicity of the essential oil (EO) from *Citrus hystrix* have been reported against *C. megacephala*, *C. rufifacies*, *L. cuprina,* and *M. domestica* [16]. In addition, plant EOs extracted from *Boesenbergia rotunda*, *Curcuma longa,* and *Ocimum gratissimum* exhibited bioactivity against insect pests. The EO of *B. rotunda* demonstrated repellent efficacy by providing 100% protection for 9 h against mosquitoes, black flies and land leeches in Thailand [17]. Insecticidal activity of *ar*-turmerone that derived from *C. longa* rhizomes yielded 100% mortality at 1000 ppm against the brown planthopper (*Nilaparvata lugens*) and diamondback moth (*Plutella xylostella*) [18]. The fumigant toxicity and repellent effect of *O. gratissimum* oil were documented against adult stored product pests, including *Sitophilus oryzae*, *Tribolium castaneum*, *Oryzaephilus surinamensis*, *Rhyzopertha dominica* and *Callosobruchus chinensis* [19]. The toxicity and oviposition deterrent effects of *Artemisia annua* and *A. dracunculus* EOs have been reported against the blow fly, *Calliphora vomitoria* [20]. EO extracted from *Piper gaudichaudianum* exhibited larvicidal activity against third instar larvae of *L. cuprina*. The results showed that these EO solubilized in ethanol or acetone, with the LD_50_ being 2.19 and 6.05 µL/cm^2^, respectively [21].

However, the inconsistent efficacy and composition of EOs, massive use of plants for high volumes of EOs, high cost of some plants, and lower potency against target pests when compared to many synthetic insecticides are problematic [22,23]. Regarding the disadvantages of insecticides and plant extracts, new enhancement strategies for both approaches are in constant demand.

The combination of two substances that performed synergism has been documented [23]. It has been reported that plant EOs have been used in combination with insecticides. For example, the deltamethrin-*Ocimum basilicum* mixture revealed synergistic action against the agricultural pest, *Spodoptera frugiperda*, by reducing 80% LD_50_ of the deltamethrin used [24]. Fazolin et al. [25] showed enhanced larvicidal activity against *S. frugiperda* with treatment of combined *Piper aduncum* oil and one of alphacypermethrin, fenpropathrin, gamma-cyhalothrin or beta-cypermethrin. The effective concentrations of these insecticides were reduced to 25–50% by the presence of the EO, when compared with administration of insecticides alone. The synergism of *Solanum xanthocarpum* with fenthion was reported against the mosquito, *Culex quinquefasciatus*, with a 1:1 ratio of LD_50_ between EO and insecticide [26]. Therefore, the aim of this study was to determine the synergistic effect of EOs with pyrethroid insecticides (permethrin and deltamethrin) against *C. megacephala*, *C. rufifacies, L. cuprina* and *M. domestica*. The combinations are expected to provide a promising alternative strategy for reducing the use of insecticides, which might be more effective than individual insecticides and plant extracts.

## 2. Materials and Methods

### 2.1. Rearing of Flies

The fly colonies of *C. megacephala*, *C. rufifacies*, *L. cuprina* and *M. domestica* were obtained from laboratory strains maintained for at least 10 years at the Department of Parasitology, Faculty of Medicine, Chiang Mai University, Thailand. They were reared in cages (30 × 30 × 30 cm) providing water and granulated sugar as food. Then, pork liver was put into the cage as a protein source and oviposition site. After female laid their eggs, approximately 100–200 first instars were transferred to transparent plastic boxes (12 × 15 × 6 cm) and fed with pork liver until the third instar reached prepupa and had no more need for feeding. The lid of each box was replaced with a fine silkscreen cloth sealed with adhesive paper tape for ventilation and prevention of larvae crawling out. The boxes were kept at natural temperatures and relative humidity until adult emergence [27].

### 2.2. Preparation of Plant EOs

**Figure 1:**
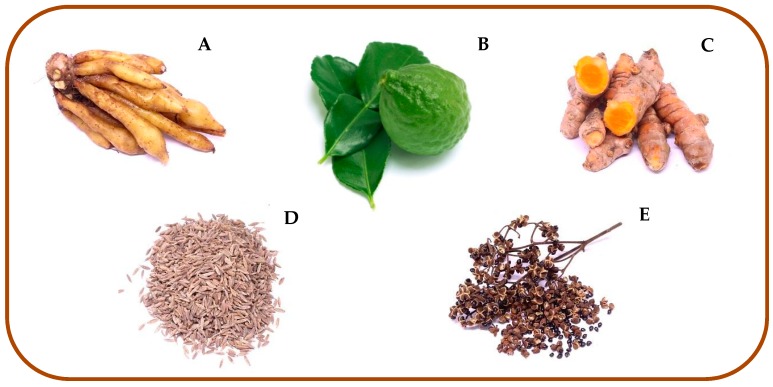
Plant material of *B. rotunda* (**A**), *C. hystrix* (**B**), *C. longa* (**C**), *O. gratissimum* (**D**), and *Z. limonella* (**E**) used in this study, purchased from herb stores in Chiang Mai province, Thailand.

Five species of plants, including finger root, *Boesenbergia rotunda* (root); kaffir lime, *C. hystrix* (fruit); turmeric, *Curcuma longa* (root); caraway, *Ocimum gratissimum* (seed) and szetchwan pepper, *Zanthoxylum limonella* (seed) (Figure 1) were purchased from a herb stores in Chiang Mai province, Thailand, and identified by a plant taxonomist at the Department of Pharmaceutical Sciences, Faculty of Pharmacy, Chiang Mai University, Thailand. Voucher specimens were deposited at the Department of Parasitology, Faculty of Medicine, Chiang Mai University.

Each plant was removed manually, shade dried in ambient temperature for 5–10 days, and ground to a coarse powder. Then, the powder was distilled following the protocol of Champakaew et al. [28]. About 250–300 g of ground plant powder was placed in an extraction column connected to a distillation flask containing ~1600 mL of distilled water and 10–15 glass beads. The distilled water was boiled to 100 °C in an immersion heater to produce steam that passed through the plant materials. The distillated mixture of essential oil and water was collected and allowed to settle into 2 layers over 3–5 days with the EO on top of the water. The water was released slowly, so that only the EO remained. The EO was dried over anhydrous sodium sulfate (Na_2_SO_4_) for 24 h and kept at 4 °C until used.

### 2.3. Gas Chromatographic-Mass Spectrometry Analysis

EOs were analyzed by gas chromatography-mass spectrometry (GC-MS) using a Hewlett-Packard GC-MS system (Model 7890). The analysis was equipped with a split-splitless injector and HP5 mass-selective detector (MSD) (30 m × 0.25 mm ID × 0.25 µm film thickness), coupled with a mass spectrometer selective detector 5975. The column temperature was increased from 50 °C to 250 °C at a rate of 10 °C/min; inlet, 250 °C splitless; injection volume, 0.5 µL; helium as the carrier gas at 1.0 mL/min; injection in split mode (250:1); total run time, 24 min. The MSD 5975 Network (EI) worked with scan parameters, 30–550 amu.; MS Quadrupole, 150 °C and MS Source, 230 °C. This analysis was performed at the Science and Technology Service Center, Chiang Mai University (STSC-CMU).

### 2.4. Preliminary Screening of EOs

Screening for adulticidal toxicity of the five EOs was conducted against adult *M. domestica*. Each EO was diluted in acetone to obtain five concentrations; and acetone alone was used as a control. Each dose was given to 30 female flies (3–5 days old). The topical application method [29] was performed by applying substances (1 µL/fly) with an autopipette (Sartorius^®^, Gottingen, Germany) on the pronotum of CO_2_ anesthetized flies. Each experiment was conducted in triplicate, and fly mortality following treatment was checked after 24 h. The lethal dose (LD) was calculated by using Finney’s probit analysis [30] for analyzing the mortality response. The three most effective EOs were selected for combining with insecticides.

### 2.5. Adulticidal Bioassay of EOs

Based on preliminary adulticidal screening results, the three most effective EOs were selected for adulticidal toxicity against *C. megacephala*, *C. rufifacies* and *L. cuprina*. The experiment was performed by topical application, with the procedure being the same as that in the preliminary screening of EOs. Each experiment was conducted in triplicate and fly mortality following treatment was checked after 24 h. The LD was calculated using Finney’s probit analysis [30].

### 2.6. Adulticidal Bioassay of Insecticides

Technical grade insecticides (permethin and deltamethrin) were used for synergistic study. Permethrin (98.1% analytical standard, Pestanal^®^, Seelze, Germany) was diluted in 10 mL of acetone to obtain the stock solution of 500 ppm; while deltamethrin (99.5% analytical standard, Dr. Ehrenstorfer^®^, Augsburg, Germany) was dissolved in 10 mL of acetone to prepare stock solution of 500 ppm. Thirty females (3–5 days old) of each fly species were treated with both insecticides. Two hundred µl of each substance was diluted in acetone to a stock solution of five concentrations. Acetone alone was used as a control. Bioassay was carried out as described in Section 2.4. Fly mortality following treatment was checked after 24 h. The LD for insecticides was calculated using Finney’s probit analysis [30] for analyzing the mortality response.

### 2.7. Adulticidal Bioassays of Binary Mixtures of EOs with Insecticides

Five variable descending concentrations were prepared for each mixture. These tests were carried out to determine the synergistic/antagonistic action resulted from vary descending at 5 concentrations of insecticides (permethrin/deltamethrin) with EO at its LD_25_ value (LD_25_ values were estimated mathematically for the expected lowest mortality dose of active compounds). The variation of permethrin and deltamethrin (min-max) concentrations for combined with three EOs (LD_25_) were shown in Table 1. Then, 30 female flies/replicates were tested using topical application. The control group was treated with acetone only. Experiments were conducted in triplicate. Fly mortality following treatment was checked after 24 h. The synergistic factor (SF) for the mixed formulation was computed after calculating LD_50_ for each combination.

The SF [31] for the combined formulation was calculated after calculating the LD_50_ for each combination.

$$\mathrm{SF}=\frac{{\mathrm{LD}}_{50}\;\mathrm{of}\;\mathrm{active}\;\mathrm{substance}\;\mathrm{alone}}{{\mathrm{LD}}_{50}\;\mathrm{of}\;\mathrm{active}\;\mathrm{substance}\;\mathrm{with}\;a\;\mathrm{synergist}}$$

SF > 1, indicates synergism; SF < 1, indicates antagonism.

### 2.8. Statistical Analysis

Regarding the toxicity assays of individual substances (EOs/deltamethrin/permethrin) and the combination of insecticides with EOs, the mortality data were analyzed using LdP line Software (Ehab Mostafa Bakr, Dokki, Cairo, Egypt) to calculate the LD. The Chi-square test was used in each bioassay for the assessment of significance and the measurement of the difference between the test samples. Statistically significant results were considered at *p* < 0.05.

## 3. Results

### 3.1. Physical and Chemical Compositions of EOs

The organoleptic properties and physical characteristics of EOs were different in name, part used, odor, color, density and yield, as shown in Table 2. The percentage of EO yields, measured as yield of EO to dry weight, ranged from 0.57% (v/w) for *C. longa* to 6.00% (v/w) for *C. hystrix*.

The GC-MS analysis for the EOs is displayed in Table 3. Twenty compounds in the EO from *B. rotunda* were identified, accounting for 96.77% of the whole oil. The three highest components were δ-3-caren (35.25%), followed by alcanfor (28.08%) and methyl cinnamate (15.10%). The EO of *C. longa* contained 17 identified compounds, representing 74.73% of the EO obtained. The 3 major components were β-turmerone (51.68%), followed by α-curcumene (7.59%) and β-sesquiphellandrene (4.74%). Twenty-eight compounds were identified from the EO of *C. hystrix*, accounting for 93.28% of the whole oil, with β-pinene (26.56%) being the principal constituent, followed by α-limonene (25.94%), and sabinene (14.01%). As for *O. gratissimum*, the EO analysis presented 18 compounds, accounting for 92.84% of the whole oil, with p-cumic aldehyde (58.21%) as the main chemical compound, followed by p-cymene (10.65%) and phenylacetylcarbinol (8.46%). With regard to *Z. limonella*, 32 compounds were identified, which represented 100% of the whole oil. The main chemical constituents were dipentene (60.22%), followed by sabinene (13.07%) and O-cymol (4.72%).

### 3.2. Adulticidal Activity of EOs

*C. longa* had the most effective toxicity in the adulticidal screening experiment on *M. domestica*, with the LD_50_ being 77.01 µg/fly, followed by *O. gratissimum* (83.11 µg/fly), *B. rotunda* (103.59 µg/fly), *C. hystrix* (106.87 µg/fly) and *Z. limonella* (225.50 µg/fly) (Table 4). Therefore, the three most effective EOs—*C. longa*, *B. rotunda* and *O. gratissimum*—were selected for adulticidal activity against the three blow fly species, *C. megacephala*, *C. rufifacies* and *L. cuprina*.

Table 5 shows the results of adulticidal toxicity of the three most effective EOs against the three blow flies. The EO of *C. longa* was the most effective, with an LD_50_ of 59.83 µg/fly, 94.52 µg/fly and 129.73 µg/fly against *L. cuprina*, *C. megacephala* and *C. rufifacies*, respectively. The adulticidal LD_50_ of *O. gratissimum* oil was 68.50 µg/fly, 110.41 µg/fly, and 166.29 µg/fly against *L. cuprina*, *C. megacephala* and *C. rufifacies*, respectively. Regarding *B. rotunda* oil, the LD_50_ was 124.64 µg/fly, 207.32 µg/fly, and 249.73 µg/fly against *L. cuprina*, *C. megacephala* and *C. rufifacies*, respectively (Table 5). No mortality was observed in the control flies.

### 3.3. Adulticidal Activity of Insecticides

Permethrin was highly toxic for *M. domestica* (LD_50_ = 0.023 µg/fly), followed by *C. megacephala* (0.050 µg/fly), *L. cuprina* (0.059 µg/fly) and *C. rufifacies* (0.060 µg/fly). In contrast, deltamethrin was highly toxic for *L. cuprina* (LD_50_ = 0.009 µg/fly), followed by *C. megacephala* (0.028 µg/fly), *C. rufifacies* (0.028 µg/fly) and *M. domestica* (0.060 µg/fly) (Table 6). No mortality was observed in the control flies.

### 3.4. Adulticidal Activity of Binary Mixtures

Table 7 shows the toxicity of permethrin-EO mixtures against all of the tested flies. Only permethrin-*C. longa* mixtures exhibited synergistic action in *C. megacephala* by decreasing the LD_50_ of permethrin from 0.05 to 0.0469 µg/fly (SF = 1.07). Meanwhile, synergistic action was observed in all permethrin-EO combinations against *C. rufifacies*, by reducing the LD_50_ of permethrin from 0.061 to 0.0093 µg/fly (SF = 6.56; permethrin-*C. longa*), followed by 0.0163 µg/fly (SF = 3.68; permethrin-*B. rotunda*), and 0.0348 µg/fly (SF = 1.72; permethrin-*O. gratissimum*). A similar trend was observed in *L. cuprina*, by lowering the LD_50_ of permethrin from 0.059 to 0.0057 µg/fly (SF = 10.35; permethrin-*C. longa*), followed by 0.0063 µg/fly (SF = 9.37; permethrin-*B. rotunda*), and 0.0108 µg/fly (SF = 5.46; permethrin-*O. gratissimum*). Permethrin-*O. gratissimum* mixtures and permethrin-*C. longa* resulted in a synergistic effect against *M. domestica*, by reducing the insecticide used from 0.023 to 0.0105 µg/fly (SF = 2.19; the former mixture) and 0.0151 µg/fly (SF = 1.52; the latter mixture). No mortality was observed in the control flies.

Regarding the combinations of deltamethrin and EOs, all of the mixtures exhibited a synergistic effect on all of the fly species (Table 8). Regarding *C. megacephala*, the LD_50_ of deltamethrin was decreased in deltamethrin-*O. gratissimum* mixtures from 0.028 to 0.0167 µg/fly (SF = 1.68), followed by 0.0176 µg/fly (SF = 1.59) in deltamethrin-*C. longa*, and 0.0264 µg/fly (SF = 1.06) in deltamethrin-*B. rotunda*. In contrast, deltamethrin-*C. longa* mixtures yielded the most effective toxicity against *C. rufifacies*, followed by deltamethrin-*B. rotunda*, and deltamethrin-*O. gratissimum* by reducing the LD_50_ of deltamethrin from 0.028 to 0.0037 µg/fly (SF = 7.57), 0.0095 µg/fly (SF = 2.95), and 0.0108 µg/fly (SF = 2.59), respectively. Regarding *L. cuprina*, the synergistic effects were obtained by reducing deltamethrin alone in deltamethrin-*C. longa* (from 0.0090 to 0.0016 µg/fly, SF = 5.63), deltamethrin-*O. gratissimum* (from 0.0090 to 0.0056 µg/fly, SF = 1.61), and deltamethrin-*C. longa* (from 0.0090 to 0.0061 µg/fly, SF = 1.48). Regarding *M. domestica*, adulticidal activity was obtained by decreasing the LD_50_ of deltamethrin alone from 0.06 to 0.0089 µg/fly (SF = 6.74; deltamethrin-*O. gratissimum*), followed by 0.009 µg/fly (SF = 6.67; deltamethrin-*C. longa*), and 0.0397 µg/fly (SF = 1.51; deltamethrin-*B. rotunda*). No mortality was observed in the control flies.

## 4. Discussion

Combined toxicity of insecticides with biopesticides has been investigated increasingly in order to reduce the use of insecticides, overcome insecticide resistance, enhance toxicity and more environmental safety. Examples were given in the combination of insecticides with fungicides applied to the honey bee, *Apis mellifera* [32,33]. This study evaluated the toxicity of pyrethorid insecticides (deltamethrin and permethrin) combined with promising plant EOs against four filth fly species (*C. megacephala, C. rufifacies, L. cuprina* and *M. domestica*).

Plants display the phenomenon of allelopathy, whereby they produce or release bioactive compounds that inhibit other organisms. More than 100,000 allelochemical groups are known, e.g., alkaloids, amines, amino acids, cyanogenic compounds, glucosides, glucosinolates, non-proteins, organic acids, peptides phenolics, polyacetylenes, quinones, and terpenoids [34]. Each EO selected herein had a distinct principle component, being δ-3-caren for *B. rotunda*, β-turmerone for *C. longa* and p-cumic aldehyde for *O. gratissimum.* Monoterpene was the compound of these oils and classified as a terpene type [35]. Rice and Coats [36] reported insecticidal activity of several monoterpenoids against *M. domestica*, the red flour beetle (*Tribolium castaneum*), and the Southern corn rootworm (*Diabrotica undecimpunctata howardi*). Molecules of these terpenes can penetrate into the cuticle of insects and interact with the target site; however, some of their portions may be metabolized and neutralized by insect defense mechanisms. Eventually, the amount of terpene molecules (toxicants) reaching the target site determines the toxicity of the substance [37]. Pavela [38] investigated the insecticidal activity of 34 EOs from a range of plants and found that the most effective, with their main components, were *Mentha pulegium* (pugelone 83.3%), *Origanum compactum* (carvacrol 58.3% and thymol 12.6%), and *Pogostemon cablin* (sesquiterpenes 95.3%), which expressed toxicity against *M. domestica*. The LD_50_ of *C. longa*, *B. rotunda*, and *O. gratissimum* EO was lower in this study than permethin/deltamethrin administered alone. This was not surprising, because EOs are composed of both active and inactive compounds, while synthetic insecticides are a single purified active compound [39]. Furthermore, the efficacy of EOs depends on the geographical origin of plants and environmental conditions, which significantly influences the biological properties of EOs [40]. Regarding the adulticidal activity of insecticides, deltamethrin exhibited higher toxicity than permethrin, which is represented by lower LD_50_ values in *C. megacephala*, *C. rufifacies* and *L. cuprina*. This result was in contrast to investigations in Chiang Mai during 2015, when permethrin (LD_50_ = 0.0028 μg/fly) indicated more susceptibility than deltamethrin (LD_50_ = 0.0461 μg/fly) in *C. megacephala* [7]. The result of the analysis for *M. domestica* in this study indicated that permethrin exhibited more toxicity than deltamethrin. This agreed with a previous study that showed *M. domestica* exhibiting more susceptibility to permethrin (LD_50_ = 0.0049 μg/fly) than deltamethrin (LD_50_ = 0.1058 μg/fly) [7]. Based on permethrin *per se*, both *C. megacephala* and *M. domestica* displayed a slight increase in insecticide resistance from 2015, showing ~18-fold and ~5-fold increased resistance, respectively [7].

Management of fly populations using chemical control with insecticide could lead to developing insecticide resistance. Meanwhile, biological control with plant EOs could not yield the efficacy achieved by insecticides, but this approach is environmentally safe. The combination in this study of deltametrin with each EO (*B. rotunda*, *C. longa* and *O. gratissimum*) and permethrin-*C. longa* mixtures showed a synergistic effect in all of the fly species, whereas an antagonistic effect was seen in *C. megacephala* and *M. domestica* treated with permethrin-*B. rotunda*, and in only *C. megacephala* with permethrin-*O. gratissimum* treatment. However, the synergistic action of binary mixtures of two insecticides is rarely known [41]. The possible reason for the synergistic efficacy could be the susceptibility of insect, development of resistance, and the different chemical compositions [42]. It is probable that the observed synergism was the result of laboratory flies overwhelmed by the mixture of insecticide combined with EOs that disrupt different target sites. Pyrethroid type I (permethrin) causes presynaptic neuron repetitive discharge in insects, and pyrethroid type II (deltamethrin) causes a tonic release of transmitters which is indicative to membrane depolarization [43], whereas EO causes deterring oviposition, inhibiting growth, repelling, and mimicking juvenile hormones [8,9].

Tong and Bloomquist [39] revealed that all of the 14 EOs, including oils of camphor (*Cinnamomum camphora* L.), thyme (*Thymus serpyllum*), *Amyris* (*Amyris balsamifera* L.), lemon (*Citrus limon* L.), cedarwood (*Juniperus virginiana*), frankincense (*Boswellia carteri* L.), dill (*Anethum graveolens* L.), myrtle (*Myrtus communis* L.), juniper (*Juniperus communis* L.), black pepper (*Piper nigrum* L.), verbena (*Lippia citriodora* L.), *Helichrysum* (*Helichrysum italicum* G.), sesame (*Sesamum indicum* L.), and sandalwood (*Santalum album* L.), combined with permethrin, had an antagonistic effect against *Ae. aegypti* larvae after 24 h exposure. Due to the high hydrophobicity and affinity of permethrin with EOs, the authors hypothesized that the molecules of permethrin were dispersed into these EOs and might reduce permethrin bioavailability. Faraone et al. [44] reported that the EO of *L. angustifolia* and *Thymus vulgaris* synergized the activity of imidacloprid against the green peach aphid, *Myzus persicae*, whereas these EOs antagonized another insecticide (spirotetramat). They suggested that the insecticide mode of action or chemical/physical properties are a crucial determinate of whether or not synergism or antagonism of insecticide-EO mixtures would occur. However, the idea of increasing opportunities for the use of EOs in pest management and reducing inputs of synthetic insecticides might be possible. One study reported the synergism of *Solanum xanthocarpum* with cypermethrin against the mosquito, *Anopheles stephensi*, with a 1:1 ratio of LD_50_ between the EO and insecticide [45]. In addition, Silva et al. [24] showed the same toxicity of *Ocimum basilicum*-deltamethrin mixtures, which were relative to deltamethrin alone against the fall armyworm, *Spodoptera frugiperda*, and this combination reduced ~80% LD_50_ of the deltamethrin used. Combinations between the ethanolic extracts of *Cichorium intybus*, *Azadirachta indica, Conyza aegyptiaca, Piper nigrum, Salix safsaf* and *Sonchus oleraceus* with insecticides (chlorpyrifos, deltamethrin, flufenoxuoron and methomyl) increased the potential effect and reduced the amount of insecticides used against the larvae and adults of the mosquito, *Anopheles pharoensis* [46]. Maurya et al. [47] revealed the larvicidal activity of *Ocimum basilicum*-imidacloprid mixtures (ratio 1:1) against *An. stephensi*, with decreasing LD_50_ of the insecticide used, and this formulation was safe for the aquatic mosquito predator, *Anisops bouvieri*, and other non-target aquatic cyclops. Such information revealed that the combination of plant EOs with insecticides increases the toxic effect against insect vectors/pests. In future, more mixtures of plant EOs with insecticides (e.g., *C. longa* + *O. gratissimum* + deltamethrin) merit investigation for enhancing synergistic efficacy for filth flies.

After being administered EO of *C. hystrix*, morphological aberration of *C. megacephala, C. rufifacies, L. cuprina,* and *M. domestica* larvae exhibited deformed midgut and hindgut, vacuolated fat cells, a decreased number of nuclei in the fat cells, and degenerative nuclei [16]. Only the adulticidal toxicity of *C. longa, B. rotunda* and *O. gratissimum* EOs combined with insecticides was assessed in this study. More bionomic investigations merit further study to determine the post-exposure of flies; for example, the development of immature stages, adult longevity and survival pattern, reproductive potential, and morphological abnormality.

## 5. Conclusions

This study assessed the synergistic action of adulticidal toxicity of plant EOs combined with permethrin/deltamethrin against *C. megacephala*, *C. rufifacies, L. cuprina,* and *M. domestica*. Although this study found the antagonistic effect in some cases, the most synergistic effect was found in the combination of Permethrin-*C. longa* against *L. cuprina*, with the highest SF of 10.35 and Deltamethrin-*C. longa* against *C. rufifacies*, with the highest SF of 7.57. These EOs presented a promising role as synergists to improve the efficacy of permethrin and deltamethrin. The mode of binary mixture application (e.g., repeated exposure with different dosages) merits further investigation for facilitating fully-competent fly management.

## Figures and Tables

**Table 1:** Variation of permethrin and deltamethrin concentrations for combining with three EOs (LD_25_).

Combination [insecticides-EOs (LD_25_)]	Concentration of Permethrin (µg/Fly)	Concentration of Deltamethrin (µg/Fly)
*C. megacephala*		
Insecticide-*C. longa*	0.010–0.016	0.010–0.060
Insecticide-*B. rotunda*	0.040–0.200	0.010–0.080
Insecticide-*O. gratissimum*	0.040–0.240	0.012–0.040
*C. rufifacies*		
Insecticide-*C. longa*	0.004–0.013	0.001–0.005
Insecticide-*B. rotunda*	0.005–0.035	0.003–0.015
Insecticide-*O. gratissimum*	0.020–0.060	0.002–0.030
*L. cuprina*		
Insecticide-*C. longa*	0.004–0.013	0.001–0.003
Insecticide-*B. rotunda*	0.002–0.015	0.001–0.005
Insecticide-*O. gratissimum*	0.010–0.020	0.002–0.015
*M. domestica*		
Insecticide-*C. longa*	0.004–0.020	0.002–0.025
Insecticide-*B. rotunda*	0.020–0.160	0.005–0.080
Insecticide-*O. gratissimum*	0.002–0.030	0.002–0.030

**Table 2:** Physical characteristics and percentage yield of essential oils (EOs).

Botanical Name Species	Name	Part Used	Physical Characteristic			% Yield
			Odor	Color	Density	
Family: Zingiberaceae *Curcuma longa*	turmeric	rhizome	ginger-like	light yellow	0.94	0.57
*Boesenbergia rotunda*	finger root	rhizome	ginger-like	pale yellow	0.92	5.98
Family: Lamiaceae						
*Ocimum gratissimum*	clove basil	seed	herb-like	light yellow	0.93	2.15
Family: Rutaceae						
*Citrus hystrix*	kaffir lime	peel of fruit	orange-like	light yellow	0.86	6.00
*Zanthoxylum limonella*	szetchwan-peper	fruit	orange-like	pale yellow	0.86	5.72

**Table 3:** GC-MS analysis of the essential oils (EOs).

No.	Constituents	Percentage Composition (%)						
		RT	BR	CL	CH	OG	ZL	KI
1	O-cymene	4.540					0.31	930
2	α-pinene	4.670	0.09		1.84	0.16	2.5	940
3	camphene	4.920	1.27					958
4	β-phellandrene	5.210				0.19		978
5	sabinene	5.230			14.01		13.07	979
6	pinene	5.320				5.91		985
7	β-pinene	5.330			26.56		0.39	986
8	β-myrcene	5.400	0.1		0.94	0.25		990
9	β-cymene	5.700		0.24			1.2	1011
10	3-carene-2,5-dione	5.740					0.1	1014
11	p-cymene	5.850			0.94		0.49	1022
12	p-cymol	5.960		0.24				1030
13	O-cymol	5.970	0.21		1.62	10.65	4.72	1031
14	limonene	6.040	0.96			0.13		1035
15	α-limonene	6.060			25.94			1037
16	β-ocimene	6.070	0.03					1038
17	dipentene	6.090					60.22	1039
18	1,8-cineol	6.100		0.66		0.06		1040
19	norsabinene	6.110	7.11				1.88	1041
20	ocimene	6.230	0.75					1049
21	β-ocimene	6.240					1.22	1049
22	γ-terpinene	6.460			1.33	5.71	0.92	1064
23	α-terpinolen	6.870			0.39		0.22	1090
24	δ-carene	7.038			0.38		0.43	1111
25	isodiprene	7.044	2.4					1101
26	rose oxide	7.210			0.92			1113
27	p-menthatriene	7.420					0.22	1128
28	oxacyclohexane	7.470			0.5			1132
29	carvyl acetate	7.640					0.22	1144
30	β-citronella	7.820			1.54			1156
31	alcanfor	7.890	28.08					1161
32	D-camphene	7.990	1.21					1167
33	M-cymene	8.090	0.19			0.07		1174
34	2-carene epoxide	8.315	0.21			0.1		1188
35	γ-terpinen	8.320					2.23	1189
36	α-methylstyrene	8.380		0.19				1192
37	crypton	8.420					1.45	1195
38	norbornene	8.515			0.99		0.61	1201
39	cyclohexadienemethanol	8.526				2.13		1202
40	1,5,8-p-menthatriene	8.590					0.31	1207
41	caprylyl acetate	8.620					0.11	1209
42	1,4,8-p-menthatriene	8.850					1.44	1226
43	β-citronellol	8.870			2.12			1229
44	(E)-carveol	9.040					0.58	1239
45	p-mentha-1,5,8-triene	9.090	0.44					1243
46	mentha-1,4,8-triene carvone	9.220					1.72	1253
47	δ-3-carene	9.280	35.25			58.21		1257
48	citral	9.510	3.07					1273
49	γ-phenylbutyric acid	9.620	0.11					1280
50	phellandral	9.730					0.46	1287
51	(S)-phellandral	9.740				0.22		1288
52	2-undecanone	9.820					1.06	1293
53	(E)-beta-ocimene	9.890	0.09					1298
54	phenylacetylcarbinol	9.930				8.46		1310
55	propanol	10.380				0.07		1335
56	citronellol acetate	10.580			0.58			1350
57	calamenene	10.640			0.33			1355
58	cuminaldehyde	10.860				0.25		1371
59	cyclofenchene	10.950					0.71	1377
60	3-carene-2,5-dione	10.960			0.28			1378
61	α-copaene	11.060			2.92			1385
62	methyl cinnamate	11.160	15.1					1392
63	germacrene D	11.210			1.66			1404
64	β-caryophyllen	11.670		0.35	1.24		0.38	1433
65	α-methylnaphthalene	11.950		0.6				1454
66	α-humulene	12.140			0.34			1469
67	α-longipinene	12.366				0.17		1486
68	α-curcumene	12.370		7.59				1487
69	1(10),4(14),5-Germacratriene	12.450			0.41		0.12	1492
70	methyl undecyl ketone	12.490					0.22	1495
71	α-curcumen	12.530		2.08				1499
72	epizonarene	12.620			0.77			1506
73	β-bisabolene	12.700		1.05				1513
74	δ-cadinene	12.860			3.22			1526
75	β-sesquiphellandrene	12.920		4.74				1531
76	cadalene	13.170			0.5			1553
77	p-mentha-1,4-diene	13.210	0.1					1556
78	γ-gurjunene	13.240			0.48			1558
79	Ar-curcumene	13.280		0.47				1561
80	isolongifolene	13.640					0.25	1590
81	1,3,6,9-decatetraene	13.720					0.24	1597
82	α-amorphene	13.960				0.1		1618
83	α-cedren	14.000		0.63				1621
84	α-cedrene	14.200		0.45				1639
85	2,5-dimethoxy-3-methylnaphthalene	14.390		0.61				1655
86	β-selinene	14.560			0.53			1670
87	β-turmerone	14.630		51.68				1676
88	Ar-tumerone	14.670		2.26				1679
89	α-atlantone	15.800		0.89				1782
	Total identified		96.77	74.73	93.28	92.84	100.00	

RT = retention time, BR = *B. rotunda*, CL = *C. longa*, CH = *C. hystrix*, OG = *O. gratissimum*, ZL = *Z. limonella*, KI = Kovat index.

**Table 4:** Adulticidal activity of five EOs against *M. domestica.*

Conc. (µg/Fly)	% Mortality	Adulticidal Activity of Five EOs (µg/fly)				
		LD_25_ (LCL–UCL)	LD_50_ (LCL–UCL)	LD_99_ (LCL–UCL)	Slope ± SE	χ^2^
*C. longa*						
23.5	2.22	61.14 (56.16–65.31)	77.01 (72.79–81.17)	170.64 (150.96–202.60)	6.73 ± 0.62	5.01
47	8.89					
75.2	47.78					
94	64.44					
122.2	95.56					
*B. rotunda*						
46	2.22	76.93 (67.85–84.24)	103.59 (96.17–110.55)	289.07 (244.10–368.95)	5.23 ± 0.54	6.91
92	35.56					
119.6	71.11					
147.2	81.11					
184	96.67					
*O. gratissimum*						
46.5	15.56	59.63 (40.76–69.30)	83.11 (64.74–103.11)	261.29 (234.82–552.02)	4.68 ± 0.37	10.51
65.1	32.22					
93	45.56					
139.5	91.11					
167.4	93.33					
*C. hystrix*						
68.8	11.11	84.51 (78.67–89.79)	106.87 (101.63–112.00)	240.13 (214.28–280.38)	6.62 ± 0.55	3.33
94.6	38.89					
120.4	55.56					
146.2	83.33					
172	93.33					
*Z. limonella*						
215	46.67	157.28 (116.78–184.16)	225.50 (196.11–245.15)	781.20 (607.66–1268.08)	4.31 ± 0.71	0.59
258	57.78					
301	73.33					
344	78.89					
387	83.33					

LD_25_ = lethal dose that killed 25% of the exposed adult flies; LD_50_ = lethal dose that killed 50% of the exposed adult flies; LD_99_ = lethal dose that killed 99% of the exposed adult flies; UCL: Upper confidence limit; LCL: Lower confidence limit; χ^2^ = chi-square; Conc. = concentration; df = degrees of freedom; SE = standard error. The unit of LD is µg/fly.

**Table 5:** The adulticidal activity of three EOs against the blow flies and the house fly.

Test Species	EO	Adulticidal Activity of Three EOs (µg/Fly)				
		LD_25_ (LCL–UCL)	LD_50_ (LCL–UCL)	LD_99_ (LCL–UCL)	Slope ± SE	χ^2^ (4 df)
*C. megacephala*	*C. longa*	72.73 (65.23–78.86)	94.52 (88.28–100.42)	233.36 (202.72–285.73)	5.93 ± 0.58	4.28
*C. rufifacies*		112.12 (106.64–116.61)	129.73 (125.42–134.09)	214.59 (197.75–240.92)	10.64 ± 0.99	5.95
*L. cuprina*		42.30 (37.22–46.89)	59.83 (54.55–65.36)	197.89 (164.59–254.67)	4.48 ± 0.39	1.38
*M. domestica*		61.14 (56.16–65.31)	77.01 (72.79–81.17)	170.64 (150.96–202.60)	6.73 ± 0.62	5.01
*C. megacephala*	*B. rotunda*	165.33 (149.84–178.28)	207.32 (193.88–220.13)	452.46 (401.57–534.81)	6.86 ± 0.65	0.54
*C. rufifacies*		212.20 (202.16–220.80)	249.73 (241.08–258.85)	437.93 (400.92–494.40)	9.54 ± 0.81	5.10
*L. cuprina*		104.34 (96.92–110.43)	124.64 (118.64–130.14)	230.11 (211.41–258.43)	8.74 ± 0.77	1.17
*M. domestica*		76.93 (67.85-84.24)	103.59 (96.17–110.55)	289.07 (244.99–368.95)	5.23 ± 0.54	6.91
*C. megacephala*	*O. gratissimum*	79.27 (45.90-88.67)	110.42 (78.94-137.09)	346.45 (342.05-922.49)	4.68 ± 0.41	12.61
*C. rufifacies*		140.45 (134.08-146.22)	166.29 (160.11–173.03)	297.76 (272.43-335.05)	9.20 ± 0.71	2.63
*L. cuprina*		49.07 (42.97-54.39)	68.50 (62.69–74.04)	216.48 (185.55–266.79)	4.66 ± 0.40	7.06
*M. domestica*		59.63 (40.76-69.30)	83.11 (64.74–103.11)	261.29 (234.82–552.02)	4.68 ± 0.37	10.51

LD_25_ = lethal dose that killed 25% of the exposed adult flies; LD_50_ = lethal dose that killed 50% of the exposed adult flies; LD_99_ = lethal dose that killed 99% of the exposed adult flies; EO: Essential oil; UCL: Upper confidence limit; LCL: Lower confidence limit; χ^2^ = chi-square; df = degrees of freedom; SE = standard error. The unit of LD is µg/fly.

**Table 6:** The adulticidal activity of insecticides against the blow flies and the house fly.

Test Species	Insecticides	Adulticidal Activity of Insecticides				
		LD_25_ (LCL–UCL)	LD_50_ (LCL–UCL)	LD_99_ (LCL–UCL)	Slope ± SE	χ^2^ (4 df)
*C. megacephala*	permethrin	0.029 (0.024–0.033)	0.050 (0.044–0.056)	0.330 (0.257–0.460)	2.83 ± 0.22	0.80
*C. rufifacies*		0.042 (0.036–0.045)	0.060 (0.055–0.066)	0.238 (0.200–0.296)	3.91 ± 0.27	6.13
*L. cuprina*		0.011 (0.005–0.018)	0.059 (0.043–0.086)	17.310 (3.87–343.26)	0.94 ± 0.14	0.30
*M. domestica*		0.011 (0.007–0.015)	0.023 (0.018–0.029)	0.316 (0.216–0.572)	2.06 ± 0.24	0.94
*C. megacephala*	deltamethrin	0.007 (0.004–0.011)	0.028 (0.021–0.035)	2.621 (1.038–12.804)	1.18 ± 0.16	2.41
*C. rufifacies*		0.015 (0.012–0.017)	0.028 (0.024–0.031)	0.243 (0.178–0.373)	2.47 ± 0.21	6.00
*L. cuprina*		0.004 (0.002–0.005)	0.009 (0.024–0.031)	0.133 (0.080–0.310)	1.95 ± 0.26	0.12
*M. domestica*		0.037 (0.032–0.042)	0.060 (0.054–0.068)	0.317 (0.243–0.457)	3.23 ± 0.28	2.78

LD_25_ = lethal dose that killed 25% of the exposed adult flies; LD_50_ = lethal dose that killed 50% of the exposed adult flies; LD_99_ = lethal dose that killed 99% of the exposed adult flies; UCL: Upper confidence limit; LCL: Lower confidence limit; χ^2^ = chi-square; df = degrees of freedom; SE = standard error. The unit of LD is µg/fly.

**Table 7:** Adulticidal toxicity of permethrin combined with three EOs against the blow flies and the house fly.

		LD_50_ of Adulticidal Toxicity (95% LCL–UCL, µg/Fly)								
Fly sp.	Per	Per + *C. longa* (LD_25_)	Slope ± SE	SF (Effect)	Per + *B. rotunda* (LD_25_)	Slope ± SE	SF (Effect)	Per + *O. gratissimum* (LD_25_)	Slope ± SE	SF (Effect)
CM	0.0500 (0.044–0.056)	0.0469 (0.0409–0.0539)	2.29 ± 0.19	1.07 (S)	0.0652 (0.0539–0.0754)	2.21 ± 0.26	0.77 (A)	0.0739 (0.0624–0.0848)	2.33 ± 0.23	0.68 (A)
CR	0.0600 (0.055–0.066)	0.0093 (0.0086–0.0102)	3.67 ± 0.40	6.56 (S)	0.0163 (0.0144–0.0183)	2.51 ± 0.24	3.68 (S)	0.0348 (0.0322–0.0373)	4.16 ± 0.41	1.72 (S)
LC	0.0590 (0.043–0.086)	0.0057 (0.0048–0.0065)	2.29 ± 0.35	10.35 (S)	0.0063 (0.0056–0.0071)	2.63 ± 0.24	9.37 (S)	0.0108 (0.0091–0.012)	3.81 ± 0.61	5.46 (S)
MD	0.0230 (0.018–0.029)	0.0151 (0.0132–0.018)	2.39 ± 0.27	1.52 (S)	0.0500 (0.0445–0.0597)	2.65 ± 0.25	0.46 (A)	0.0105 (0.0084–0.0132)	1.27 ± 0.15	2.19 (S)

LD_50_ = lethal dose that killed 50% of the exposed adult flies; SF = synergistic factor; S = synergism; A = antagonism; Per = permethrin; Fly sp. = fly species; UCL = upper confidence limit; LCL = lower confidence limit; SE = standard error; CM = *C. megacephala*; CR = *C. rufifacies*; LC = *L. cuprina*; MD = *M. domestica*.

**Table 8:** Adulticidal toxicity of deltamethrin combined with three EOs against the blow flies and the house fly.

LD_50_ of Adulticidal Toxicity (95% LCL–UCL, µg/Fly)										
Fly sp.	Del	Del + *C. longa* (LD_25_)	Slope ± SE	SF (Effect)	Del + *B. rotunda* (LD_25_)	Slope ± SE	SF (Effect)	Del + *O. gratissimum* (LD_25_)	Slope ± SE	SF (Effect)
CM	0.0280 (0.021–0.035)	0.0176 (0.0145–0.0204)	2.07 ± 0.25	1.59 (S)	0.0264 (0.0215–0.0316)	1.58 ± 0.19	1.06 (S)	0.0167 (0.0151–0.0182)	3.55 ± 0.39	1.68 (S)
CR	0.0280 (0.024–0.031)	0.0037 (0.0033–0.0042)	2.56 ± 0.30	7.57 (S)	0.0095 (0.0087–0.0105)	3.43 ± 0.33	2.95 (S)	0.0108 (0.0093–0.0127)	1.97 ± 0.18	2.59 (S)
LC	0.0090 (0.024–0.031)	0.0016 (0.0015–0.0018)	3.47 ± 0.39	5.63 (S)	0.0061 (0.0049–0.0087)	2.00 ± 0.32	1.48 (S)	0.0056 (0.0045–0.0069)	1.52 ± 0.21	1.61 (S)
MD	0.0600 (0.054–0.068)	0.0090 (0.0075–0.0108)	1.58 ± 0.17	6.67 (S)	0.0397 (0.0333–0.0489)	1.81 ± 0.18	1.51(S)	0.0089 (0.0072–0.0109)	1.41 ± 0.16	6.74 (S)

LD_50_ = lethal dose that killed 50% of the exposed adult flies; SF = synergistic factor; S = synergism; A = antagonism. Del = deltamethrin; Fly sp. = fly species; UCL = upper confidence limit; LCL = lower confidence limit; SE = standard error; CM = *C. megacephala*; CR = *C. rufifacies*; LC = *L. cuprina*; MD = *M. domestica*.
